# NPA-Cu^2+^ Complex as a Fluorescent Sensing Platform for the Selective and Sensitive Detection of Glyphosate

**DOI:** 10.3390/ijms22189816

**Published:** 2021-09-10

**Authors:** Fang Sun, Xin-Lu Ye, Yu-Bo Wang, Ming-Li Yue, Ping Li, Liu Yang, Yu-Long Liu, Ying Fu

**Affiliations:** 1 Department of Chemistry, College of Arts and Sciences, Northeast Agricultural University, Harbin 150030, China; sunfangsunxue@163.com (F.S.); wangyubo970629@163.com (Y.-B.W.); yueml@neau.edu.cn (M.-L.Y.); liping198904@163.com (P.L.); yangliu@neau.edu.cn (L.Y.); liuyulong@neau.edu.cn (Y.-L.L.); 2 Department of Applied Biology and Chemical Technology, Hong Kong Polytechnic University, Hung Hom, Hong Kong, China; 18082612d@connect.polyu.hk

**Keywords:** fluorescent sensor, NPA-Cu^2+^, glyphosate, coordination competition, off-on

## Abstract

Glyphosate is a highly effective, low-toxicity, broad-spectrum herbicide, which is extensively used in global agriculture to control weeds and vegetation. However, glyphosate has become a potential threat to human and ecosystem because of its excessive usage and its bio-concentration in soil and water. Herein, a novel turn-on fluorescent probe, N-n-butyl-4-(3-pyridin)ylmethylidenehydrazine-1,8-naphthalimide (**NPA**), is proposed. It efficiently detected Cu^2+^ within the limit of detection (LOD) of 0.21 μM and displayed a dramatic turn-off fluorescence response in CH_3_CN. **NPA-Cu^2+^** complex was employed to selectively and sensitively monitor glyphosate concentrations in real samples accompanied by a fluorescence turn-on mode. A good linear relationship between **NPA** and Cu^2+^ of glyphosate was found in the range of 10–100 μM with an LOD of 1.87 μM. Glyphosate exhibited a stronger chelation with Cu^2+^ than **NPA** and the system released free **NPA** through competitive coordination. The proposed method demonstrates great potential in quantitatively detecting glyphosate in tap water, local water from Songhua River, soil, rice, millet, maize, soybean, mung bean, and milk with mild conditions, and is a simple procedure with obvious consequences and no need for large instruments or pretreatment.

## 1. Introduction

Glyphosate (*N*-(phosphonomethyl) glycine) is an efficient, low-toxicity, and nonselective herbicide against perennial weeds [1]. It is widely used in economic crops or trees such as orchards, rice, maize, soybeans, wheat, and tea fields [2,3,4]. However, glyphosate has become a risk to human health because of its improper treatment and its bioconcentration in soil and water [5]. Glyphosate is a potential endocrine disruptor and exhibits adverse effects on cell cycle regulation [6,7]. It is classified as a potential carcinogen and genotoxic to humans by the International Agency for Research on Cancer (IARC) [8]. The U.S. Environmental Protection Agency set a maximum concentration of 700 μg/L for glyphosate in water [9]. Guidelines for Canadian Drinking Water Quality prescribe a limit of 280 μg/L in drinking water [10]. Therefore, establishing a convenient and reliable method for the accurate and rapid detection of glyphosate is necessary and of great urgency.

Several effective analytical strategies have been established for the detection of glyphosate. Traditional methods depend on expensive large-scale equipment, such as gas chromatography (GC), GC coupled with mass spectrometry (GC-MS), and high performance liquid chromatography (HPLC) [11,12]. To overcome the limitations of expensive instruments, tedious pretreatments, and derivatization procedures, several techniques have been proposed, such as enzyme-linked immunosorbent assay (ELISA) [13], capillary electrophoresis (CE) [14], amperometry [15], colorimetric assay [16], fluorescence spectrometry, and electrochemical sensing [17,18]. These analytical techniques overcome the disadvantages of traditional methods to a certain extent; however, there are still some limitations. For example, ELISA utilizes expensive antibodies that are sensitive to temperature and pH, and electrochemical assays generally have short life cycles. Colorimetry is obvious; however, lacks good accuracy. Hence, it is critical to develop a facile, rapid, sensitive, and efficient method to detect glyphosate in environmental samples.

**Scheme 1:**
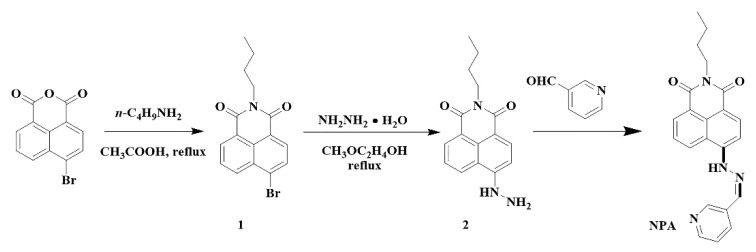
Synthesis route to compound **NPA**.

Recently, fluorescence detection for glyphosate has attracted much attention because of its easy operation, rapid response, high sensitivity, etc. Fluorescence sensors such as nanoclusters [19,20,21], quantum dots [22,23,24], and metal-organic frameworks have been used to detect glyphosate [25]. Currently, copper ion complexes are widely used due to their novelty, sensitivity, low toxicity, and membrane permeability [26,27,28]. A coumarin derivative and Cu^2+^ complex system was developed that selectively detected glyphosate with a good linear relationship of 0.02–1.50 μg/mL [29]. Inspired by the designs of copper complexes and our continuous interest in pesticide identification [30,31,32,33,34], a new probe for Cu^2+^, *N*-n-butyl-4-(3-pyridin)ylmethylidenehydrazine-1,8-naphthalimide (**NPA**), was designed and synthesized (Scheme 1). **NPA** exhibited fluorescence quenching in the presence of Cu^2+^. When glyphosate was added to the **NPA-Cu^2+^** system, the fluorescence was discovered with a color change from colorless to green. **NPA-Cu^2+^** can be used for ultra-sensitive detection of glyphosate in environmental samples with high sensitivity, fast response, and obvious consequences.

## 2. Results

### 2.1. Spectral Characteristics of Probe **NPA** upon Coordination with Cu^2+^

The solvent effects of **NPA** were studied in solvents of CH_3_CN, CH_3_CH_2_OH, DMSO, DMF, and CH_3_OH, and CH_3_CN was selected as the test solvent (Figure S4).

**Figure 1:**
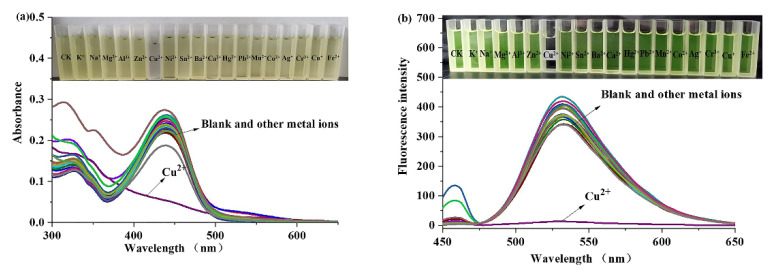
UV–vis (**a**) and fluorescence (**b**) spectra of **NPA** toward various metal cations.

The responses of **NPA** were examined to evaluate the sensing properties with the introduction of other different metal ions. The UV–vis absorption spectrum of free **NPA** showed a maximum peak at 440 nm. The absorbance intensity of **NPA** decreased, accompanied by the yellow solution of **NPA** changing to colorless after the addition of Cu^2+^, while the UV–vis spectra of other metal ions basically did not change, which indicated that **NPA** could serve as a highly selective “naked-eye” probe for Cu^2+^ (Figure 1a). The introduction of Cu^2+^ into the solution of **NPA** also caused significant fluorescence quenching (Figure 1b). Free **NPA** exhibited strong fluorescence with emission at 533 nm. The fluorescence intensity decreased quickly and was quenched with Cu^2+^ added after 100 s, after which the system color changed from green to colorless (Figure S5).

To further explore the characteristics of **NPA** for Cu^2+^, competition and reversibility experiments were carried out. The fluorescence intensity of the **NPA-Cu^2+^** system was slightly enhanced after Al^3+^ was added, and the introduction of other interference metal ions did not affect the detection of Cu^2+^ by **NPA** (Figure S6). Alternating additions of Cu^2+^ and EDTA to the system caused variation in the fluorescence intensity (Figure S7). Although a certain degree of decrease in the fluorescence intensity was found after several cycles, **NPA** showed good reversibility. The results indicate that **NPA** could serve as a specific, rapid, and reversible sensor for Cu^2+^ detection.

**Figure 2:**
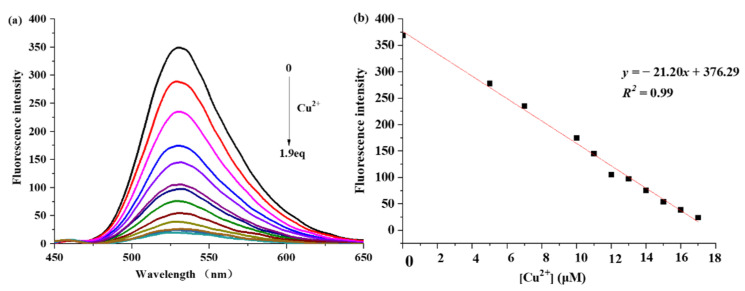
(**a**) Fluorescence spectrum of **NPA** on the addition of different amounts of Cu^2+^ in a CH_3_CN solution; (**b**) the linear responses of **NPA** with Cu^2+^ concentrations.

The fluorescence intensity of **NPA** was recorded at various concentrations of Cu^2+^ in CH_3_CN for quantitative analysis. The fluorescence intensity decreased gradually with increasing the Cu^2+^ concentration from 0 to 1.9 eq. and remained stable after 1.9 eq. Cu^2+^ was added (Figure 2a). A desirable linear relationship was observed between the fluorescence intensity and Cu^2+^ concentration, represented by *y* = −21.20*x* + 376.29 (*R^2^* = 0.99) (Figure 2b). The binding constant (*K*_11_) was calculated to be 1.8 × 10^4^ M^−1^ (Figure S8), which was calculated with validated open-source software (BindFit) [35]. The limit of detection (LOD) for Cu^2+^ was calculated to be 0.21 μM based on the equation of LOD = 3*σ/k*, where *σ* is the blank standard deviation and *k* is the slope of the fluorescence intensity ratio vs. analyte concentration plot [36]. Comparing with **NPA** and other Cu^2+^ sensors based on Schiff’s base with different sensing mechanisms (Table S1), the LOD of **NPA** was low enough to detect Cu^2+^.

**Figure 3:**
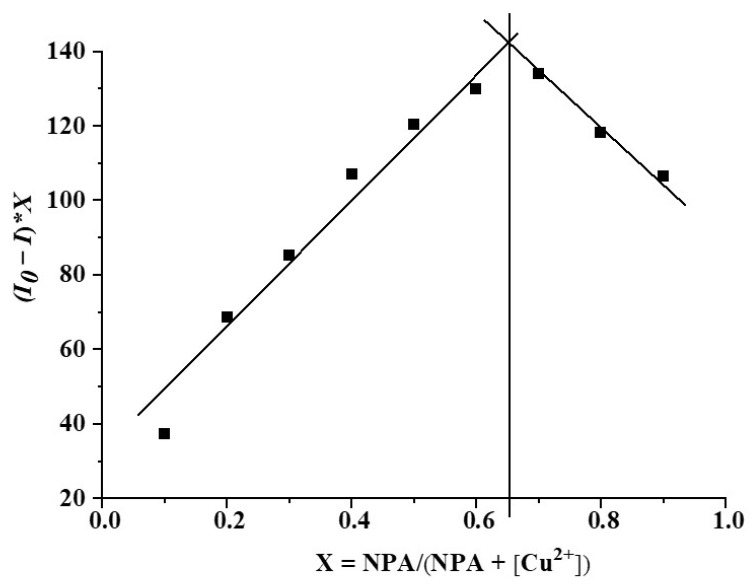
Job’s plot for the binding ratio between **NPA** and Cu^2+^.

To determine the stoichiometric ratio of **NPA** binding to Cu^2+^, a Job’s plot was constructed (Figure 3). The maximum fluorescence change was observed at a mole fraction of 0.65, indicating a 2:1 binding mode of **NPA** and Cu^2+^.

**Figure 4:**
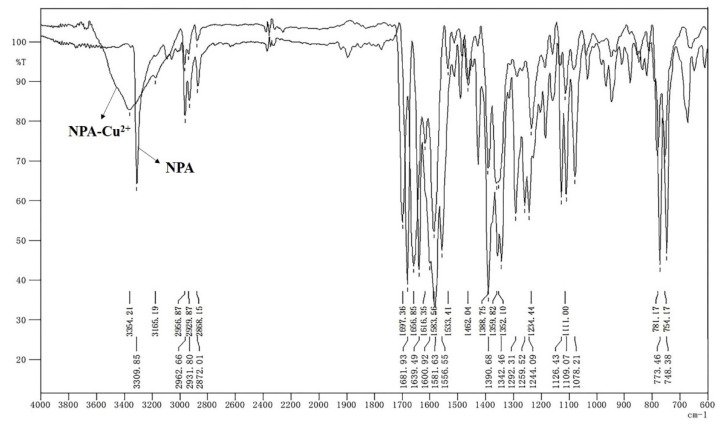
The FT-IR spectra of **NPA** compound before and after the introduction of Cu^2+^.

**Figure 5:**
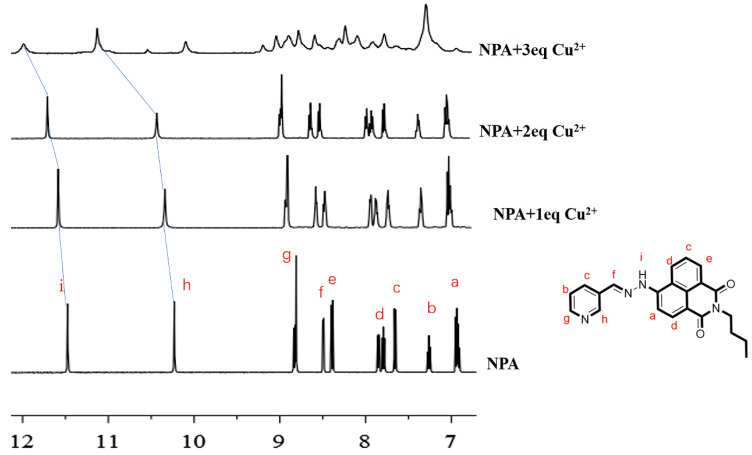
^1^H NMR titration plots of **NPA** probe with Cu^2+^ in DMSO-*d*_6_. (a, c, d, e: naphthalimide; b, c, g, h: pyridine; f: CH-N; i: NH.

**Scheme 2:**
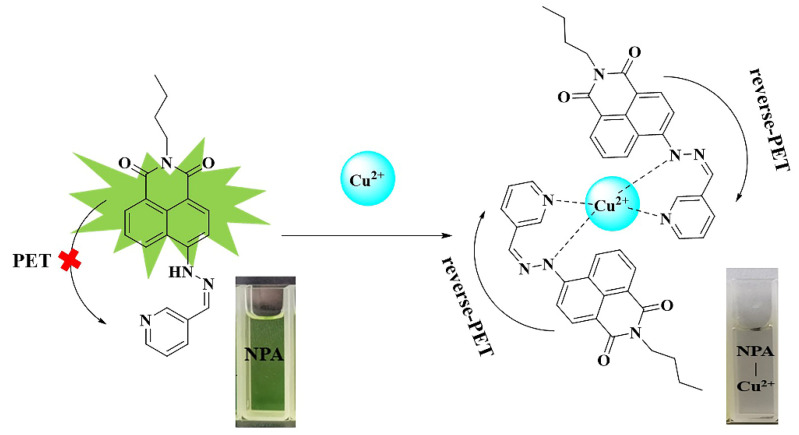
Possible sensing mechanism of **NPA** and Cu^2+^.

The IR and NMR spectra of **NPA-Cu^2+^** were employed to elucidate the sensing mechanism. The IR spectroscopic comparison is shown in Figure 4 and Figure 5. These results further confirm that NH participated in the complex leading to a reverse photoinduced electron transfer (PET) process and resulted in fluorescence quenching (Scheme 2).

**Figure 6:**
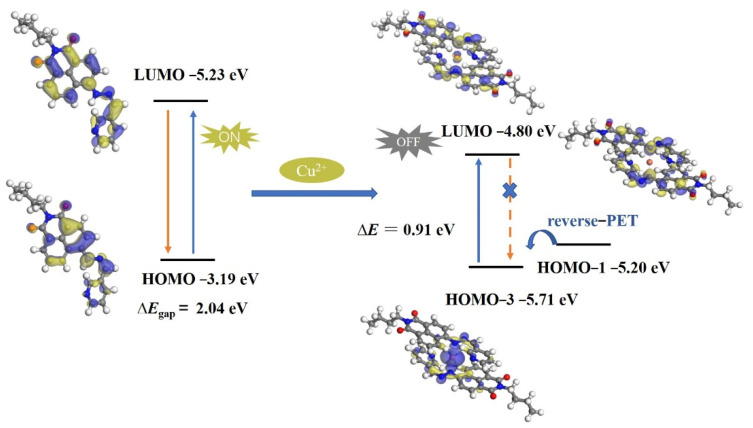
Frontier orbital energy diagram and electron transfer path in **NPA** and **NPA-Cu^2+^**.

To further explore the changes in **NPA** and Cu^2+^ before and after coordination, the frontier orbital energy diagram of **NPA** and **NPA-Cu^2+^** from the PBE was constructed using DFT (Figure 6). Electrons of the fluorophore in **NPA** transferred from its HOMO to the LUMO and then returned to its HOMO without passing through the HOMO of the receptor in **NPA**, causing the free probe **NPA** to exhibit a fluorescence when on. When Cu^2+^ was added, the Δ*E* of the system decreased, and the electrons were transferred from the 1,8-naphthalimide moiety (HOMO-1) to the electrondeficient pyridine groups (HOMO-3). These results demonstrate that the quenching response of **NPA** to Cu^2+^ ions could be considered a reverse PET process [37,38,39].

### 2.2. Sensing Assay for Glyphosate by **NPA-Cu^2+^**

**Scheme 3:**
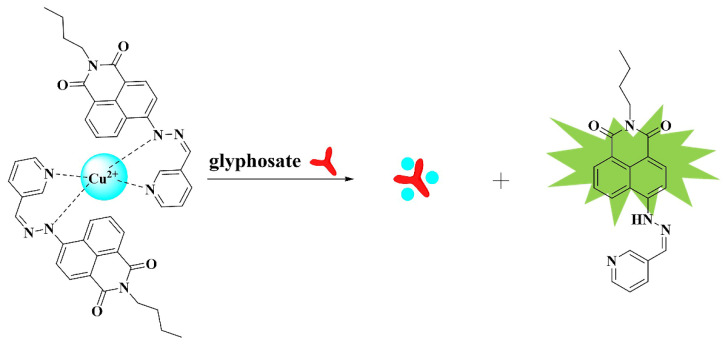
Proposed sensing mechanism of **NPA-Cu^2+^** and glyphosate.

The binding mode between glyphosate and Cu^2+^ has been confirmed by EXAFS spectra [40,41]. Cu^2+^ ions lie at the center of a Jahn-Teller distorted octahedron with the amine, carboxylate, and phosphonate groups of glyphosate chelating with Cu^2+^ to form two five-membered chelate rings oriented in the equatorial plane. A sensing approach for glyphosate was developed, inspired by the aforementioned mechanism via competitive coordination between the **NPA-Cu^2+^** complex and glyphosate, as shown in Scheme 3. When glyphosate is present in the **NPA-Cu^2+^** system, the functional groups of glyphosate, such as carboxylate, amine, and phosphonate, chelated with Cu^2+^ to form the more stable complex, in order to release the **NPA**, and the fluorescence intensity of the system can recover. Therefore, it is feasible in theory to regard the competitive coordination balance between **NPA-Cu^2+^** and glyphosate as a new method for the detection of glyphosate residue.

**Figure 7:**
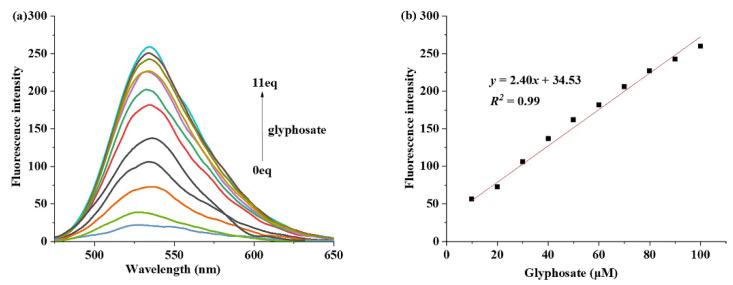
(**a**) Fluorescence spectrum of **NPA-Cu^2+^** on the addition of different amount of glyphosate in a CH_3_CN solution; (**b**) the linear responses of **NPA-Cu^2+^** with glyphosate concentrations.

The fluorescence intensity gradually increased with increasing glyphosate concentration in the **NPA-Cu^2+^** system (Figure 7a). The fluorescence response versus the concentrations of glyphosate showed a desirable linear relationship in the concentration range of 0 to 11 eq., as described by the linear equation *y* = 2.40*x* + 34.53, with a linear coefficient of 0.99 (Figure 7b). The binding constant (*K*_11_) was calculated as *K*_11_ = 9.6 × 10^4^ M^−1^, according to Host-Guest interaction BindFit (Figure S9). The LOD was calculated to be 1.87 μM (0.32 μg/mL).

**Scheme 4:**
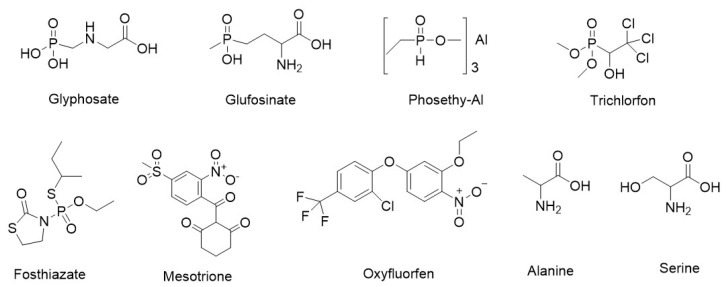
The structures of test interferences.

**Figure 8:**
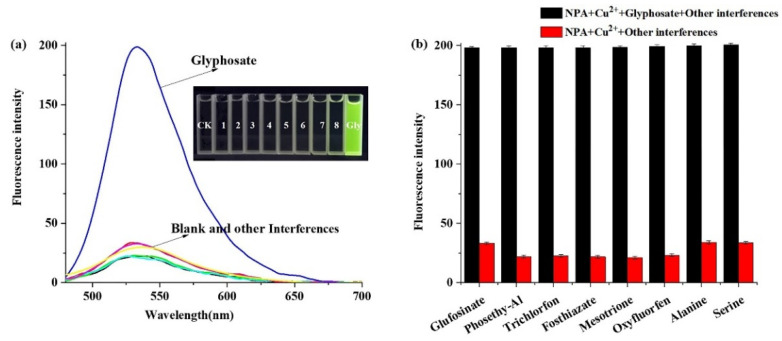
(**a**) Fluorescence spectral changes in **NPA-Cu^2+^** toward various pesticides (Gly: glyphosate); (**b**) fluorescence response of **NPA-Cu^2+^** upon the addition of various pesticides.

To evaluate the specificity of the **NPA-Cu^2+^** system to glyphosate, selective and interferential tests were conducted. Some typical organophosphorus pesticides were selected, such as glufosinate, trichlorfon, phosethy-Al, trichlorfon, and fosthiazate. In addition, the triketone herbicide mesotrione, the fluorine-containing pesticide oxyfluorfen, and common everyday ligands (alanine and serine) were selected as negative interferences (Scheme 4). As shown in Figure 8a, after introducing glyphosate to the **NPA-Cu^2+^** system, the fluorescence intensity of the system increased significantly. When glufosinate, alanine, and serine were added, the fluorescence intensity of the system slightly increased, while the other pesticides did not affect the chelation (Figure 8b).

Other pesticides did not interfere with **NPA-Cu^2+^** system, and glyphosate was quickly and specifically recognized within 300 s (Figure S10). All of these results indicate that **NPA-Cu^2+^** detects glyphosate specifically and quickly, and is valuable in monitoring glyphosate in real time.

### 2.3. Applications in Real Samples

To explore the practicality of the **NPA-Cu^2+^** system, tap water, local water from Songhua River, soil collected from the Northeast Agricultural University campus (Harbin, China), rice, millet, maize, soybean, mung bean, and milk directly purchased from local supermarkets were used for comparison. Different concentrations (30, 60, and 90 μM) of glyphosate standard solutions were added to these samples and detected through the **NPA-Cu^2+^** system. Satisfactory fortified recoveries of 98.8–116.7% were obtained, and the relative standard deviations were all less than 2.5% (Table 1). Compared with previously reported sensing systems for glyphosate (Table 2), this strategy is more sensitive than others, and features no enzymatic mild conditions, has no need for large instruments or pretreatment, but has a simple procedure with obvious consequences [42,43,44,45]. The results indicate that the sensor system possesses significant potential for practical applications related to the environmental monitoring of glyphosate.

## 3. Discussion

### 3.1. Sensing Mechanism of Cu^2+^ by **NPA**

There was a sharp peak at around 3309 cm^−1^ in the IR spectra of **NPA**, which could be assigned to the NH of the hydrazine group. This peak was replaced by a broad peak at 3358 cm^−1^ in the complex (**NPA-Cu^2+^**) spectrum, indicating the involvement of NH in the complexation. To further investigate the binding site of **NPA** with Cu^2+^, ^1^H NMR titrations were carried out. The obvious change was that the proton signal (site i) of the NH gradually decreased with the addition of Cu^2+^, while the proton signal of the =CH− group in the pyridine ring at 10.23 ppm (site h) gradually shifted to the lower field due to the paramagnetic properties of Cu^2+^. When 3 eq. Cu^2+^ was added, the proton signal of the NH almost disappeared and the proton signal of the =CH− group in the pyridine ring moved to 10.85 ppm. These further confirm that NH participated in the complex leading to a reverse photoinduced electron transfer (PET) process and resulted in fluorescence quenching [46,47,48].

### 3.2. Sensing Mechanism of Glyphosate by **NPA-Cu^2+^**

The experimental result that the **NPA-Cu^2+^** complex system can specifically recognize glyphosate is attributed to the relative spatial proximity of the phosphonate amino and carboxyl groups of glyphosate to each other, facilitating the formation of chelates with Cu^2+^. Although glufosinate and glyphosate are similar in structure, the amino and phosphonate groups are separated by three methylenes in glufosinate, which make it difficult to form a steric chelation with Cu^2+^. Alanine and serine are structurally similar to glufosinate, and cannot form a stable steric chelation with Cu^2+^. However, other similar pesticides lacking coordination groups failed to form stable coordinations with Cu^2+^, and could not form chelations in space.

## 4. Materials and Methods

### 4.1. Materials and Physical Instruments

All analytical reagent-grade chemicals and solvents employed for the experiment, which were purchased from commercial providers, were used without further purification. Glyphosate, glufosinate, trichlorfon, phosethy−Al, fosthiazate, mesotrione, and oxyfluorfen were purchased from Altai Biological Technology Co., Ltd. (Hebei, China).

FT−IR spectra were measured on a Bruker ALPHA−T spectrometer, which used KBr pellets with a range of 4000–600 cm^−1^ (Bruker Corp., Billerica, MA, USA). The ^1^H and ^13^C NMR spectra were recorded on a Bruker AV400 NMR spectrometer by using DMSO-*d*_6_ as the solvent, and the chemical shifts are reported in ppm (Bruker Corp., Billerica, MA, USA). The HRMS was obtained on an FTMS Ultral Apex MS spectrometer (Bruker Corp., Billerica, MA, USA). The absorption spectra were gained on a Shimadzu UV-2700 UV–vis spectrometer at 25 °C (Shimadzu Corp., Kyoto, Japan). Fluorescence spectra were obtained on a PerkinElmer LS55 fluorescence spectrometer (PerkinElmer Corp., Waltham, MA, USA) with a xenon lamp and quartz carrier.

### 4.2. Synthesis of the Probe NPA

N-n-butyl-4-bromo-1,8-naphthalimide (1) and N-n-butyl-4-hydrazine hydrate-1,8 naphthalic anhydride (2) were synthesized according to previous research of our group [49,50,51]. Compound 2 (283.0 mg, 1 mmol) and β-nicotinaldehyde (40%, *w/w*, 3.0 mL) were added to EtOH (20 mL). The mixture was refluxed for 2 h and then cooled to room temperature. The precipitate was filtered and washed with EtOH to obtain an orange solid with a yield of 66%. IR (KBr, ν, cm^−1^) 3309(N–H), 2957, 2858(C–H), 1635(C=O), 1126(C–N). ^1^H NMR (400 MHz, DMSO-*d*_6_) δ 11.48 (s, 1H), 10.23 (s, 1H), 8.82 (d, J = 5.9 Hz, 1H), 8.49 (d, J = 7.3 Hz, 1H), 8.39 (d, J = 8.5 Hz, 1H), 7.85 (d, J = 7.8 Hz, 1H), 7.82–7.77 (m, 1H), 7.66 (d, J = 8.5 Hz, 1H), 7.26 (t, J = 7.7 Hz, 1H), 6.96–6.90 (m, 2H), 4.06–4.01 (m, 2H), 1.65–1.57 (m, 2H), 1.35 (m, J = 7.4 Hz, 2H), 0.93 (t, J = 7.4 Hz, 3H).^13^C NMR (100 MHz, DMSO-*d*_6_) δ 164.02, 163.36, 149.94, 148.27, 146.53, 140.92, 134.01, 133.69, 131.31, 131.22, 129.45, 128.61, 125.50, 124.62, 122.46, 119.13, 112.00, 107.65, 40.02, 30.23, 20.31, 14.19. HRMS (ESI): calculated for C_22_H_20_N_4_O_2_ [M + H]^+^ 373.1658, obtained a value of 373.1659.

The original spectra of **NPA** are included in the electronic Supplementary Information (Figures S1–S3).

### 4.3. General Procedures for Spectrophotometric Studies

The solvent effects of **NPA** (10^−5^ M) were studied in the solvents of CH_3_CN, CH_3_CH_2_OH, DMSO, DMF, and CH_3_OH, and then CH_3_CN was selected as the solvent to be tested. The stock solutions of 10^−2^ M metal ions were provided from NaCl, KCl, CuCl, AgNO_3_, CuCl_2_, MgCl_2_, NiCl_2_, ZnCl_2_, SnCl_2_, BaCl_2_, MnCl_2_, CaCl_2_, HgCl_2_, PbCl_2_, BaCl_2_, CoCl_2_, HgCl_2_, PbCl_2_, FeCl_2_, AlCl_3_, and CrCl_3_ using ultrapure water. A Cu^2+^ solution (1 mL, 10^−2^ M) was added to a 10 mL volumetric flask and diluted to 10^−3^ M in ultrapure water. **NPA** (10^−5^ M) solutions of 9.0, 8.0, 7.0, 6.0, 5.0, 4.0, 3.0, 2.0, and 1.0 mL were taken and transferred to 10.0 mL volumetric flasks. Cu^2+^ (10^−3^ M) solutions of 10, 20, 30, 40, 50, 60, 70, 80, and 90 µL were added to each **NPA** solution, so that the mole fractions of **NPA** became 0.1, 0.2, 0.3, 0.4, 0.5, 0.6, 0.7, 0.8, and 0.9, respectively. Each bottle was diluted with CH_3_CN to 10 mL [52]. Ethylene diamine tetraacetic acid (EDTA) solution (10^−2^ M) for a reversible experiment was obtained with ultrapure water.

### 4.4. Theoretical Calculation Methods

The quantum chemical calculations of the optimized structures were conducted by applying density functional theory (DFT) in Material Studio. The structure was optimized using the Perdew-Burke-Ernzerh (PBE) method of generalized gradient approximation (GGA) in the Dmol3 module for **NPA** and a model **NPA-Cu^2+^** complex.

### 4.5. Measurement of Glyphosate Using the **NPA-Cu^2+^** System

An **NPA** solution (10.0 mL, 10^−4^ M) and a Cu^2+^ aqueous solution (200 μL, 10^−2^ M) were added to a 100 mL volumetric flask, and then the volume was fixed with CH_3_CN to configure the **NPA-Cu^2+^** solution (10^−5^ M). Subsequently, the final mixture was incubated for 5 min at 30 °C, and the fluorescence intensity at 533 nm was recorded with λ_ex_ at 440 nm. Interference (glyphosate, glufosinate, trichlorfon, phosethy-Al, fosthiazate, mesotrione, oxyfluorfen, alanine, and serine) solutions (10^−2^ M) were prepared for interference and selective experiments.

### 4.6. Applications in Real Samples

In order to verify the practical applications, tap water, local water from Songhua River, and soil collected from the Northeast Agricultural University campus (Harbin, China), rice, millet, maize, soybean, mung bean, and milk purchased directly from the local supermarkets were selected as the real samples and were tested by the proposed sensor through the standard addition method. Water from Songhua River was filtered through a 0.22 µm membrane to remove large solids and most impurities. The rice, millet, maize, soybean, and mung bean samples were ground into powders. Soil and rice millet, maize, soybean, and mung bean samples (10 g) were dispersed in 100 mL deionized water under ultrasonication for 15 min, centrifuged at 5000 rpm for 10 min, and then filtered through a 0.22 μm microporous membrane for subsequent analyses [40,53]. Additionally, different concentrations (30, 60, and 90 μM) of glyphosate standard solutions were added to these samples and detected through the **NPA-Cu^2+^** system.

## 5. Conclusions

In summary, a highly selective and sensitive naphthalimide-based derivative sensor **NPA** and a Cu^2+^ complex (**NPA-Cu^2+^** system) for glyphosate detection were established. **NPA** was synthesized as an indicator of Cu^2+^ with turn-off fluorescence through coordination, and the LOD for Cu^2+^ detection was found to be 0.21 μM. The phosphonate, amino, and carboxyl groups of glyphosate are relatively close to each other, allowing a chelate to form, and thus the space cyclization to chelate with Cu^2+^. When glyphosate was added to the **NPA-Cu^2+^** system, **NPA** was released within 300 s and the fluorescence showed a turn-on pattern with a detection limit of 1.87 μM for glyphosate. Further study revealed that glyphosate was successfully detected in water, soil, rice, millet, maize, soybean, mung bean, and milk samples by the **NPA-Cu^2+^** system. Considering the merits of the **NPA-Cu^2+^** system to detect glyphosate, namely it is rapid, sensitive mild, easy to operate, and does not require large-scale equipment, it is expected to become a potential method for the efficient detection of glyphosate.

## Figures and Tables

**Table 1:** Detection of glyphosate in water samples and soil samples using the proposed method.

Samples	Added (μM)	Found (*n* = 3) (μM)	Recovery (*n* = 3) (%)	RSD (%)
Soil	- 30	0.0 ± 0.0 34.5 ± 0.1	- 115.0 ± 1.7	- 1.9
	60	62.9 ± 0.2	104.8 ± 0.4	1.2
	90	93.6 ± 0.1	104.0 ± 0.1	2.0
	-	0.0 ± 0.0	-	-
Songhua River	30	35.2 ± 0.3	116.7 ± 0.2	2.5
	60	66.0 ± 0.1	110.3 ± 0.1	1.5
	90	94.1 ± 0.6	104.4 ± 1.1	0.9
	-	0.0 ± 0.0	-	-
Tap Water	30	33.2 ± 0.2	109.6 ± 1.1	1.6
	60	65.4 ± 0.4	105.6 ± 0.1	1.4
	90	89.3 ± 0.5	98.8 ± 0.1	2.1
	-	0.0 ± 0.0	-	-
	30	32.5 ± 0.1	108.3 ± 0.8	1.5
Rice	60	62.8 ± 0.3	104.6 ± 0.3	2.3
	90	89.5 ± 0.5	99.4 ± 0.5	1.8
	-	0.0 ± 0.0	-	-
	30	31.9 ± 0.3	106.3 ± 1.3	1.6
Milk	60	61.7 ± 0.5	102.8 ± 1.5	1.1
	90	89.8 ± 0.6	99.7 ± 0.8	2.3
	-	0.0 ± 0.0	-	-
	30	31.5 ± 0.9	105.0 ± 0.3	0.9
Millet	60	61.4 ± 0.7	102.5 ± 0.1	0.5
	90	90.6 ± 0.9	100.7 ± 0.1	0.7
	-	0.0 ± 0.0	-	-
	30	30.7 ± 0.3	102.3 ± 0.3	0.7
Maize	60	60.7 ± 0.2	101.2 ± 0.1	0.5
	90	90.5 ± 0.3	105.5 ± 0.1	0.7
	-	0.0 ± 0.0	-	-
	30	30.6 ± 0.2	102.3 ± 0.3	0.9
Soybean	60	60.8 ± 0.4	101.5 ± 0.5	1.0
	90	90.2 ± 0.3	100.5 ± 0.6	1.8
	-	0.0 ± 0.0	-	-
	30	29.0 ± 0.4	96.6 ± 0.1	0.4
Mung bean	60	59.2 ± 0.3	98.6 ± 0.6	0.5
	90	89.4 ± 0.2	99.3 ± 0.3	0.3

**Table 2:** Comparison of different strategies to detect glyphosate.

Sensing System	Range (μg/mL)	LOD (μg/mL)	Application	Reference
CDs–Fe^3+^	0.1–16	8.75	Potatoes Water Serum Soil Water	[22]
Gold Electrode	50–300	2.0		[42]
LC-MS/MS	1–250	0.5		[43]
Electrochemical sensor	1.7–16.9	0.98		[44]
Capillary electrophoretic methodologies	1.0–8.0	0.5		[45]
**NPA-Cu^2+^**	0–18.6	0.32	Water Soil	This work

## Data Availability

Not applicable.

## Supplementary Materials

The following are available online at https://www.mdpi.com/article/10.3390/ijms22189816/s1.
