# Supplementary material for: NPA-Cu2+ Complex as a Fluorescent Sensing Platform for the Selective and Sensitive Detection of Glyphosate
Source: Int J Mol Sci. 2021 Sep 10;22(18):9816. doi: 10.3390/ijms22189816 (PMC8469908; doi:10.3390/ijms22189816)
Supplement: Supplementary file 1 [file ijms-22-09816-s001.zip › ijms-1370876-Supplementary.pdf]

International Journal of *Molecular Sciences*

MDPI

Supplementary Materials

1. The Spectra of Structural Characterization of NPA

^{1}H NMR (400 MHz, DMSO-d_6) δ 11.48 (s, 1H), 10.23 (s, 1H), 8.82 (d, *J* = 5.9 Hz, 1H), 8.49 (d, *J* = 7.3 Hz, 1H), 8.39 (d, *J* = 8.5 Hz, 1H), 7.85 (d, *J* = 7.8 Hz, 1H), 7.82 – 7.77 (m, 1H), 7.66 (d, *J* = 8.5 Hz, 1H), 7.26 (t, *J* = 7.7 Hz, 1H), 6.96 – 6.90 (m, 2H), 4.06 – 4.01 (m, 2H), 1.65 – 1.57 (m, 2H), 1.35 (m, *J* = 7.4 Hz, 2H), 0.93 (t, *J* = 7.4 Hz, 3H).^{13}C NMR (100 MHz, DMSO–d_6) δ 164.02, 163.36, 149.94, 148.27, 146.53, 140.92, 134.01, 133.69, 131.31, 131.22, 129.45, 128.61, 125.50, 124.62, 122.46, 119.13, 112.00, 107.65, 40.02, 30.23, 20.31, 14.19. HRMS (ESI): calculated for $C_{22}H_{20}N_4O_2$ $[M + H]^+$ 373.1658, found 373.1659.

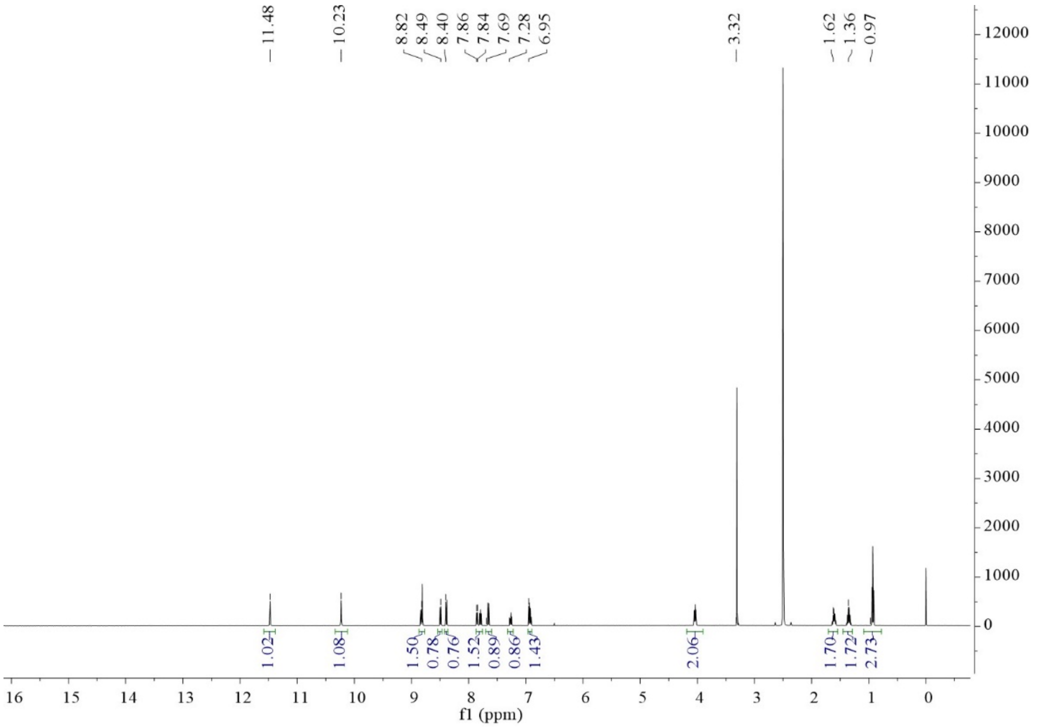

Figure S1. ^{1}H NMR spectrum of compound ***NPA***.

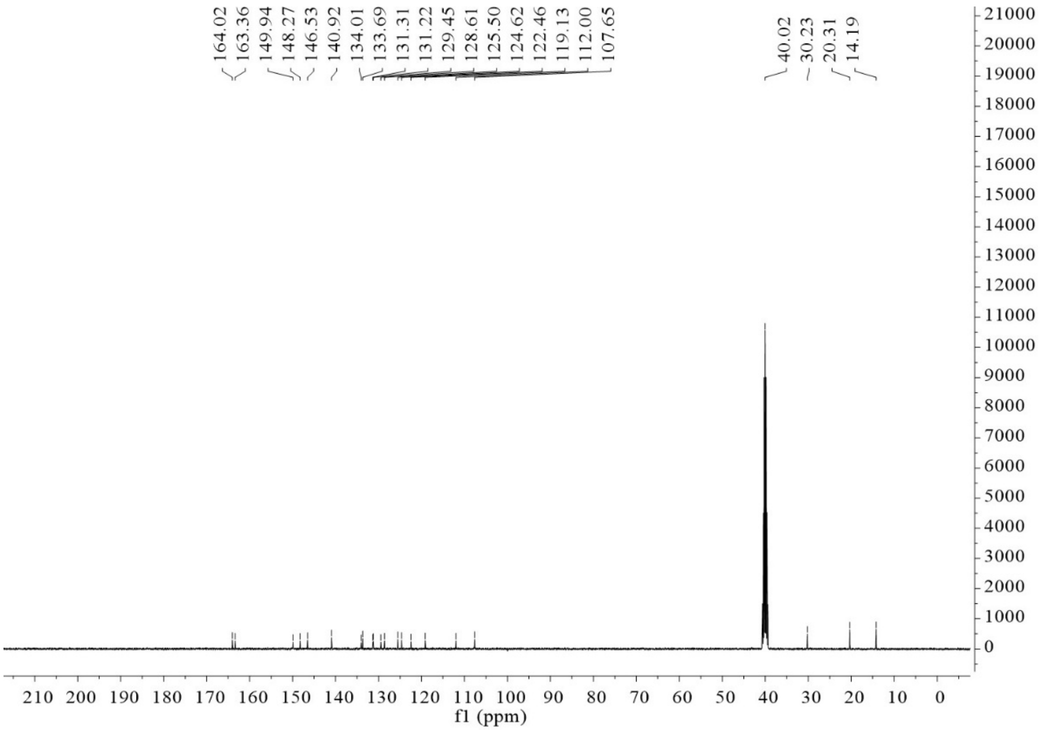

Figure S2. ^{13}C NMR spectrum of compound ***NPA***.

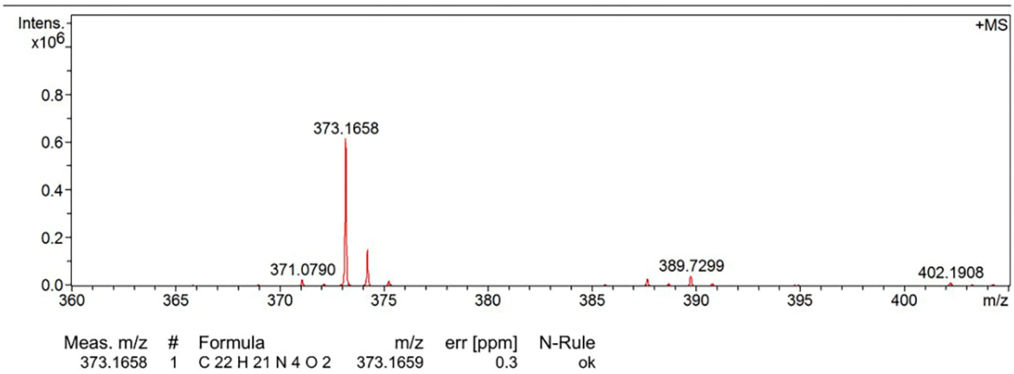

Figure S3. Mass Spectrum ($M+H^+$) of compound ***NPA***.

2. Spectral Characteristics of Probe NPA upon Coordination with Cu^{2+}

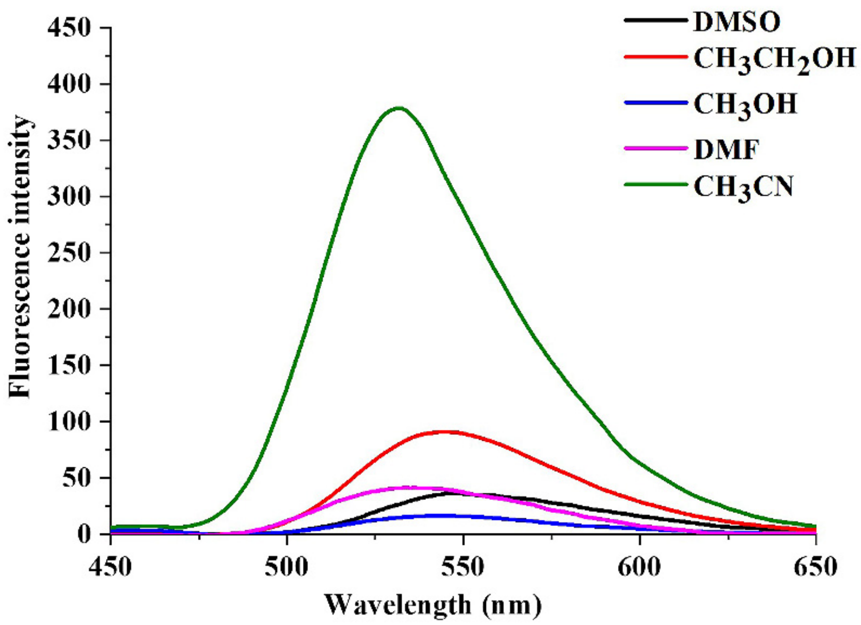

Figure S4. Fluorescence spectra of **NPA** in different solvents.

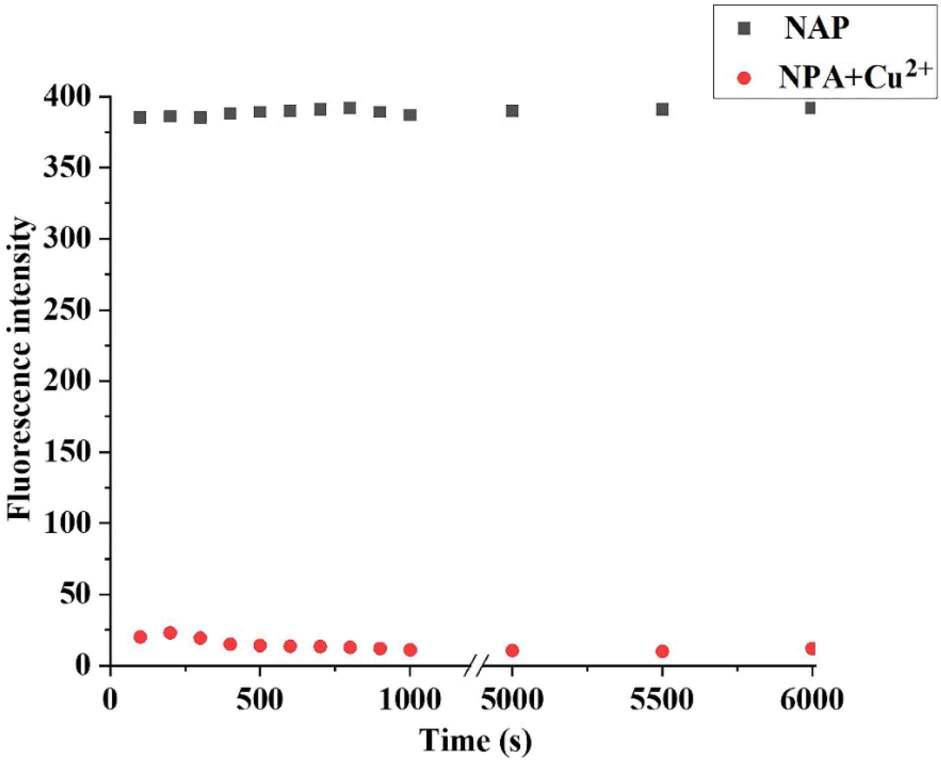

Figure S5. Stability of time on fluorescence intensity of **NPA.**

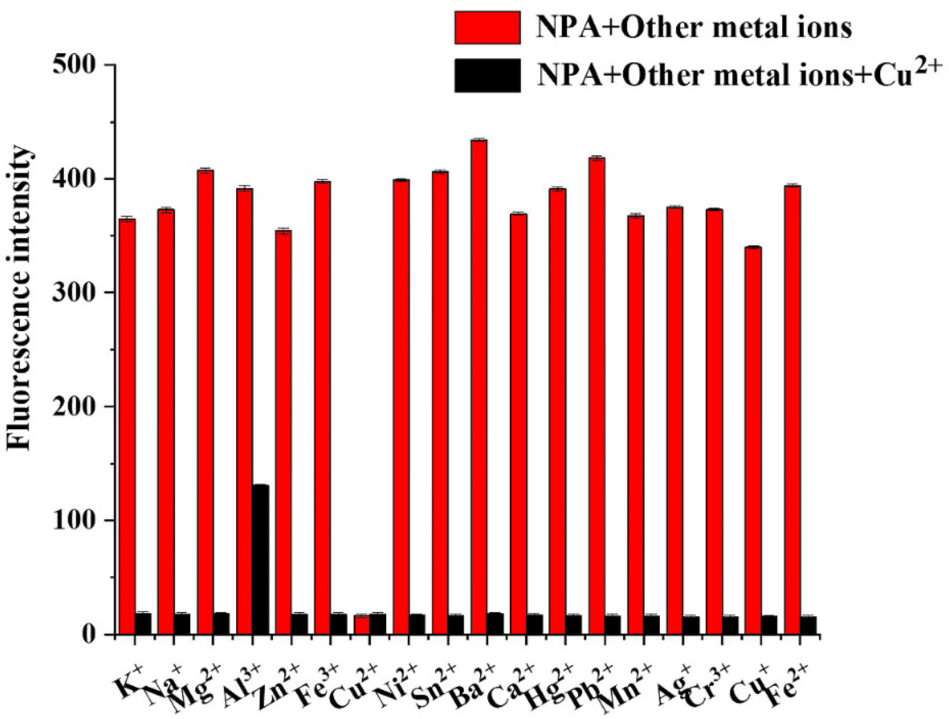

Figure S6. Fluorescence response of **NPA–Cu^{2+}** by adding various metal ions.

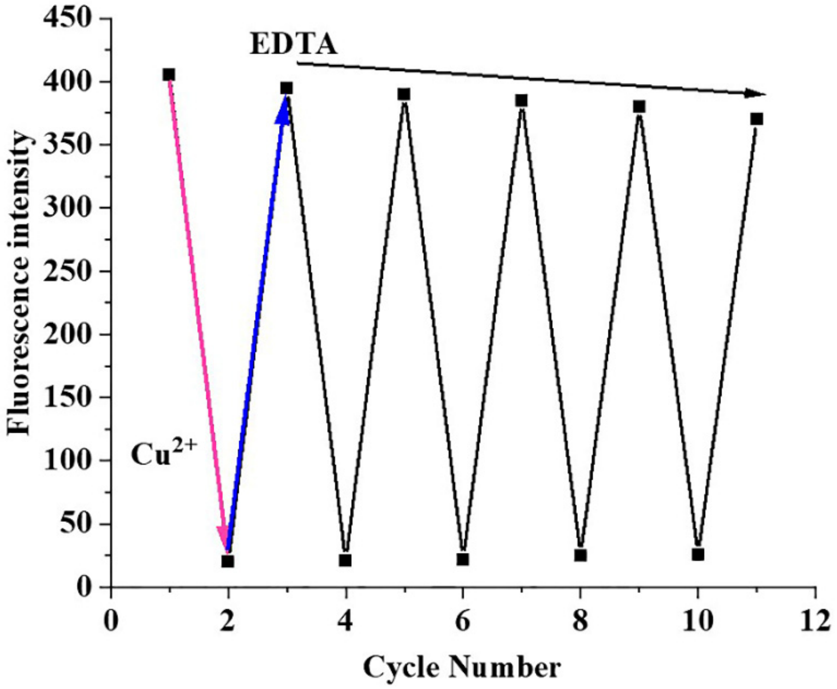

Figure S7. Reversible changes in the fluorescence intensity upon the sequential introduction of Cu^{2+} and EDTA in CH_3CN.

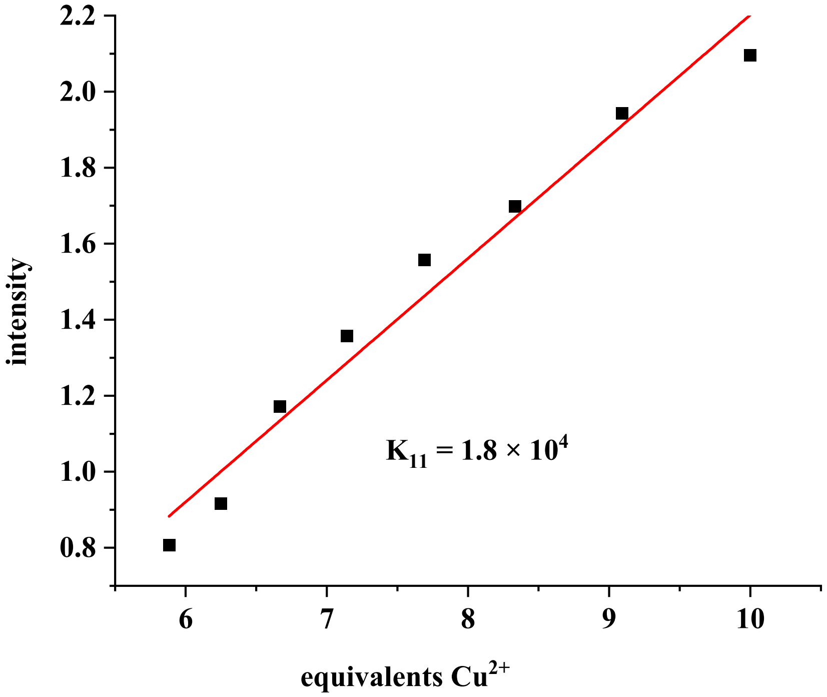

Figure S8. Host-Guest interaction BindFit of **NPA** and Cu^{2+}.

3. Sensing Assay for Glyphosate by *NPA-Cu*²⁺

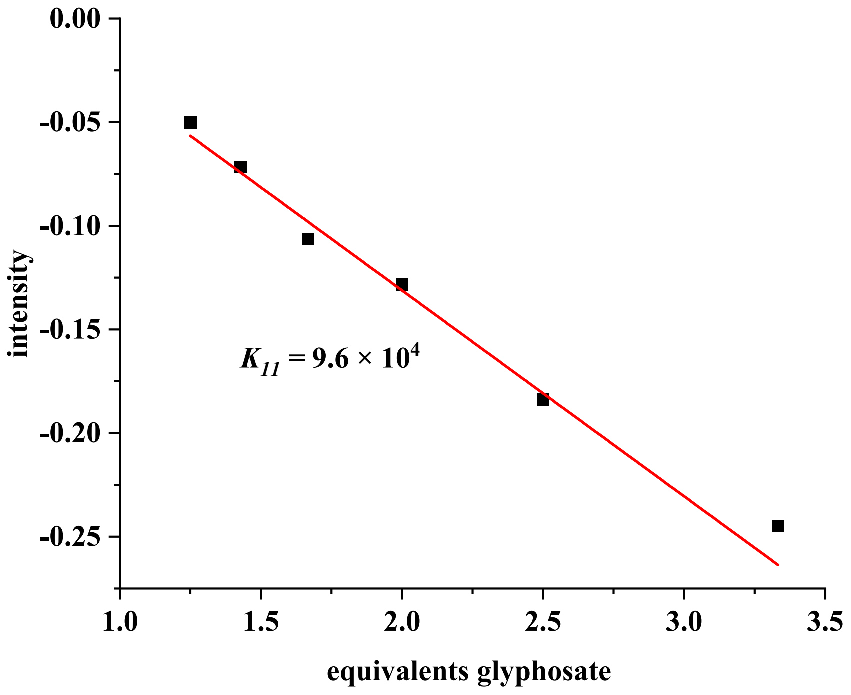

Figure S9 Host-Guest interaction BindFit of **NPA−Cu**$^{2+}$ and glyphosate.

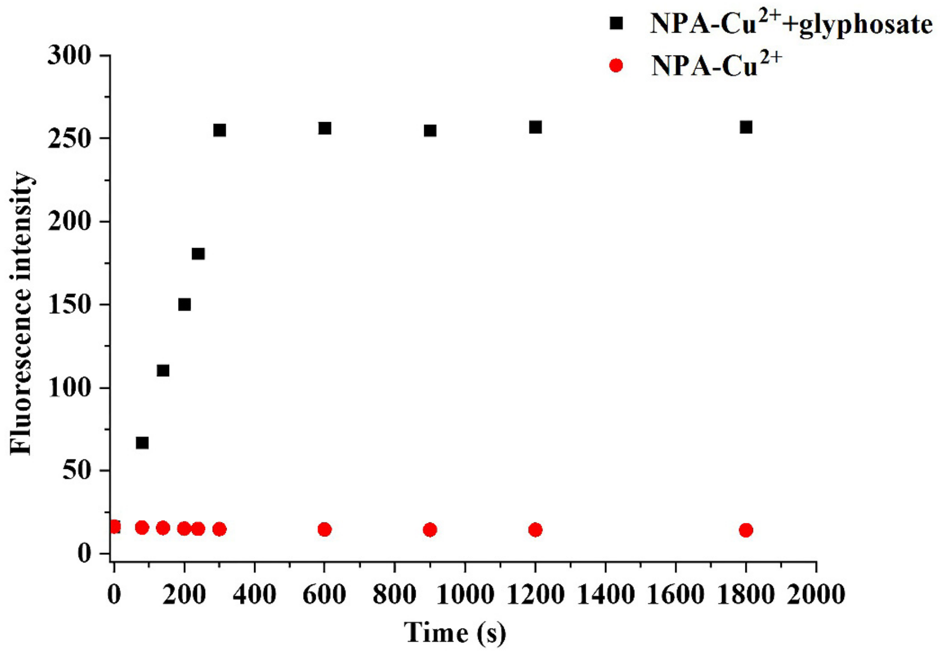

Figure S10. Stability of time on fluorescence intensity of **NPA-Cu**$^{2+}$.

4. Comparison of *NPA* with Previously Published Probes for Cu^{2+} Ions

Table S1. Comparison of **NPA** with previously published probes for Cu^{2+} ions.

Sensing Mechanism	Probes	Working Media	LOD (μM)	References
Hydrolysis	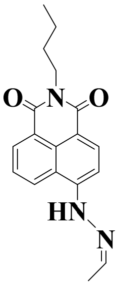	CH_3CN-HEPES (4:1, v/v, pH = 7.4)	0.32	[35]
ICT	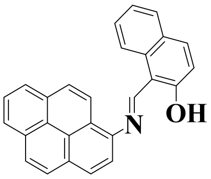	DMSO/H_2O (v/v ,8/2)	0.22	[36]
ICT	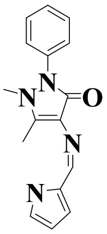	CH_3CN-H_2O (*v*/*v* = 2:8, pH = 5)	0.22	[37]
PET	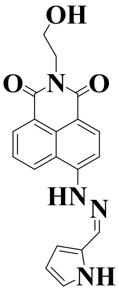	CH_3CN-H_2O (*v*/*v* = 3:7, pH = 7.0)	0.71	[43]
PET	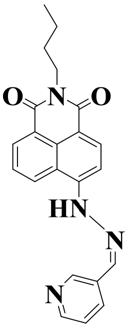	CH_3CN	0.21	This work

References

35. Fu, Y.; Pang, X.-X.; Wang, Z.-Q.; Chai, Q.; Ye, F. A highly sensitive and selective fluorescent probe for determination of Cu (II) and application in live cell imaging. *Spectrochim. Acta Part A: Mol. Biomol. Spectrosc.* **2019**, *208*, 198–205, doi:10.1016/j.saa.2018.10.005.
36. Bhorge, Y.R.; Tsai, H.-T.; Huang, K.-F.; Pape, A.J.; Janaki, S.N.; Yen, Y.-P. A new pyrene-based Schiff-base: A selective colorimetric and fluorescent chemosensor for detection of Cu(II) and Fe(III). *Spectrochim. Acta Part A: Mol. Biomol. Spectrosc.* **2014**, *130*, 7–12, doi:10.1016/j.saa.2014.03.110.
37. Xiong, J.-J.; Huang, P.-C.; Zhang, C.-Y.; Wu, F.-Y. Colorimetric detection of Cu^{2+} in aqueous solution and on the test kit by 4-aminoantipyrine derivatives. *Sensors Actuators B: Chem.* **2016**, *226*, 30–36, doi:10.1016/j.snb.2015.11.113.
43. Bao, Z.; Qin, C.; Wang, J.-J.; Sun, J.; Dai, L.; Chen, G.; Mei, F. A sensitive and selective probe for visual detection of Cu^{2+} based on 1, 8-naphthalimidederivative. *Sensors Actuators B: Chem.* **2018**, *265*, 234–241, doi:10.1016/j.snb.2018.03.050.
